# Re-Evaluating the Role of Robotic Gait Training in Post-Stroke Balance Rehabilitation

**DOI:** 10.3390/brainsci15050427

**Published:** 2025-04-22

**Authors:** Rocco Salvatore Calabrò

**Affiliations:** IRCCS Centro Neurolesi “Bonino-Pulejo”, Cda Casazza, SS 113, 98123 Messina, Italy; roccos.calabro@irccsme.it

The latest meta-analysis conducted by Loro et al. provides a valuable in-depth review of robotic gait training (RAGT) and its impact on balance rehabilitation in stroke survivors [1]. Stroke remains one of the leading causes of disability in the world, and balance impairment is one of the most challenging consequences for survivors, often leading to decreased mobility and an increased risk of falling. Traditional therapies have been used for decades to counteract these effects, but with recent technological advances in robotics, new therapies like RAGT [2] have been developed. However, as can be seen from Loro’s study, there are no solid data to demonstrate that RAGT is more effective than traditional therapy, especially in recovering balance. This research, which compiles and pools data collected from a total of 18 different randomized clinical trials, presents results that offer evidence for the modest advantage of Robotic Assisted Gait Training (RAGT) over traditional therapy interventions. Specifically, there is a notable mean difference indicated on the Berg Balance Scale (BBS), with statistical results showing a pMD of 2.17 and a 95% Confidence Interval that ranges from 0.79 to 3.55. On the other hand, while the results from the Timed Up and Go test (TUG) also lean towards favoring the outcomes of the RAGT results, we observe that these results did not reach statistical significance [1].

Notably, meta-regression analyses indicate that the duration of treatment is a significant element in enhancing TUG performance, placing emphasis on the role of session duration in rehabilitation planning. While Loro’s findings provide support for the concept that RAGT can be beneficial, they also point out the need for patient-specific variables and the individualization of treatments. Perhaps the most significant areas that require additional research and analysis (considering balance as the main outcome measure) involve the fundamental distinction between end-effector robotic devices and exoskeleton robotic devices. These two categories have shown extensive usage and application within the rehabilitation sphere [3]. End-effector devices, for instance, the G-EO System, operate by relocating the patient’s feet along an exact predetermined pathway. This mechanism provides required external assistance, while simultaneously allowing some degree of variability and flexibility in the patient’s movement patterns. These sophisticated devices have been shown to be especially useful in greatly improving gait symmetry and weight shifting, which are essential elements for successful balance recovery according to reference [4]. On the other hand, exoskeletons, such as popular models like the Lokomat and Ekso Bionics models, provide a more structured and rigid body-worn device that significantly aids in limb movement through the action of direct mechanical support. While exoskeletons offer more proprioceptive feedback and more normal weight-bearing, their stiffness sometimes limits the variability of motion needed to maximize gait patterns. The study by Loro et al. does not differentiate these two categories of robotic devices clearly in its findings [1], but this distinction is relevant when tailoring rehabilitation programs to the individual needs of the patient [5].

Another factor that is worthy of note in the conscientious deliberation of RAGT as a pertinent subject of debate is its profound effect on neuroplasticity—the brain’s amazing ability to rewire itself by forming new neural pathways throughout one’s life.

One of the most significant advantages of RAGT is that it has an amazing capacity to induce changes in neuroplasticity in the brain that can effectively support and facilitate motor learning [6,7]. This is achieved via the repetitive, high-intensity, and task-specific execution of movements that are specially tailored to the needs of the patient. RAGT, or robot-assisted gait training, provides a predictable and consistent platform for practicing movement. This predictability of practice is necessary to maximize the formation of neural connections and induce the reorganization of the motor networks following the occurrence of a stroke [6]. Some evidence has demonstrated that robotic-assisted gait training, or RAGT, has a significant ability to induce more cortical reorganization in rehabilitation patients. This potential is heightened particularly when RAGT is combined with real-time feedback systems and functional electrical stimulation, which work together to create an enhanced therapy regimen. This understanding supports and confirms the fact that RAGT is not merely a mechanical aid device, but one that actively participates in the overall neurological rehabilitation process.

That being said, it should be understood that the universal adaptation and widespread application of RAGT are confronted by numerous obstacles that can impede forward movement, including issues related to costs and access for individuals who might potentially benefit from this new technology. Robotic rehabilitation equipment is extremely costly, not only when buying the machines, but also when servicing and training staff [8].

The majority of centers, particularly in middle- and low-income countries, are not well equipped to have such technologies as part of routine clinical practice.

Even in well-funded healthcare systems, these technologies are primarily available to specialist clinics, thereby creating disparities in rehabilitative access. The cost-effectiveness of RAGT is still a debatable topic, with the high up-front and ongoing costs needing to be balanced out against the long-term gains in terms of enhanced patient outcomes, caregiver burden reduction, and possible reductions in hospitalization and fall-related injury [9]. Additionally, insurance for RAGT varies greatly across various healthcare systems, with the majority of patients being unable to afford the out-of-pocket expenses for robotic therapy. There are also ethical considerations regarding whether limited resources should be allocated for expensive robotic devices when traditional methods of rehabilitation can provide equivalent outcomes in the majority of cases. In addition, logistical concerns like the requirement for specially trained staff members to run these systems and the physical limitations of rehabilitation centers create further obstacles [9]. While RAGT has the ability to revolutionize stroke rehabilitation, these issues of cost and accessibility must first be addressed to enable the greater dissemination of its benefits.

It is noteworthy that the prospective contribution of RAGT to balance in neurological patients extends beyond mobility improvement [10]. With repetitive, controlled motion, RAGT has the potential to enhance postural control, weight transfer, and proprioceptive feedback, all of which are prerequisites for balance recovery. Additionally, the incorporation of real-time feedback and adaptive resistance into robotic devices can further increase neuroplasticity and generate even more pronounced motor relearning.

Since impaired balance is a significant contributor to increased fall risk and loss of independence in stroke survivors and those suffering from other neurological disorders, this impairment should be the focus of further research [10]. Future research studies should aim to refine RAGT protocols with the specific goal of targeting impairments in balance, including multisensory integration, task-specific training, and therapy adaptations that are individually tailored. An improved understanding of the impact of RAGT on balance mechanisms would translate to more efficient rehabilitation methods, and consequently, a better quality of life and functional independence for patients with neurological disorders. The systematic review by Loro et al. provides an urgently needed contribution to the ongoing debate on the efficacy of RAGT in the balance rehabilitation of post-stroke survivors [1]. While the data are strongly suggestive of the existence of potential benefits from these treatments, especially when they are appropriately combined with the standard therapy methods, it is imperative for more research to be conducted to address a variety of key questions. These involve questions regarding optimal patient selection, the individualization of treatment regimens, and the determination of differential effects that can result from the use of different robotic modalities. In addition to these concerns, it is necessary to tackle the issues of accessibility and cost. It is essential to address these considerations so that the technological innovation we see in the field of rehabilitation does not unintentionally play a role in increasing the level of existing healthcare disparities between populations [9].

In conclusion, as robotic rehabilitation continues to grow and evolve as a discipline, it is becoming even more clear that a concerted, collaborative effort across a number of disciplines, including engineering, neuroscience, and clinical practice, to name a few, will be of utmost importance in attempts to fully capitalize upon and utilize the enormous potential that robotic rehabilitation has in benefiting those recovering from stroke. This systematic review has successfully established the baseline foundation for this immensely exciting next phase of research and development. In this significant study, the paramount importance of employing robotic interventions specifically tailored to balance is accentuated, emphasizing their key function in developing this line of inquiry.
